# Regulation of cardiomyocyte intracellular trafficking and signal transduction by protein palmitoylation

**DOI:** 10.1042/BST20221296

**Published:** 2024-02-22

**Authors:** Kobina Essandoh, James P. Teuber, Matthew J. Brody

**Affiliations:** 1 Department of Pharmacology, University of Michigan, Ann Arbor, MI, U.S.A.; 2 Division of Cardiovascular Medicine, Department of Internal Medicine, University of Michigan, Ann Arbor, MI, U.S.A.

**Keywords:** cardiomyocyte, exocytosis, intracellular signaling, palmitoylation, S-acylation, trafficking

## Abstract

Despite the well-established functions of protein palmitoylation in fundamental cellular processes, the roles of this reversible post-translational lipid modification in cardiomyocyte biology remain poorly studied. Palmitoylation is catalyzed by a family of 23 zinc finger and Asp-His-His-Cys domain-containing S-acyltransferases (zDHHC enzymes) and removed by select thioesterases of the lysophospholipase and α/β-hydroxylase domain (ABHD)-containing families of serine hydrolases. Recently, studies utilizing genetic manipulation of zDHHC enzymes in cardiomyocytes have begun to unveil essential functions for these enzymes in regulating cardiac development, homeostasis, and pathogenesis. Palmitoylation co-ordinates cardiac electrophysiology through direct modulation of ion channels and transporters to impact their trafficking or gating properties as well as indirectly through modification of regulators of channels, transporters, and calcium handling machinery. Not surprisingly, palmitoylation has roles in orchestrating the intracellular trafficking of proteins in cardiomyocytes, but also dynamically fine-tunes cardiomyocyte exocytosis and natriuretic peptide secretion. Palmitoylation has emerged as a potent regulator of intracellular signaling in cardiomyocytes, with recent studies uncovering palmitoylation-dependent regulation of small GTPases through direct modification and sarcolemmal targeting of the small GTPases themselves or by modification of regulators of the GTPase cycle. In addition to dynamic control of G protein signaling, cytosolic DNA is sensed and transduced into an inflammatory transcriptional output through palmitoylation-dependent activation of the cGAS-STING pathway, which has been targeted pharmacologically in preclinical models of heart disease. Further research is needed to fully understand the complex regulatory mechanisms governed by protein palmitoylation in cardiomyocytes and potential emerging therapeutic targets.

## Introduction

Cysteine palmitoylation or S-acylation is the reversible attachment of saturated fatty acids onto protein cysteine thiols that functions as a critical regulatory mechanism to modulate protein function by facilitating targeting to cellular membrane microdomains [1–3]. Palmitoylation consequently can impact intracellular trafficking, protein stability, and protein–protein interactions to exert dynamic control of intracellular signal transduction, secretory pathway activity, and/or cellular physiology [1,2,4–6]. Despite the increasing recognition of a central role for palmitoylation in regulating fundamental homeostatic and pathophysiological cellular processes, its functions in cardiomyocytes, the major cardiac cell type responsible for the contractile activity of the heart and predominant culprit in most idiopathic and inherited forms of cardiomyopathy and heart failure [7–9], remain poorly studied. Palmitoylation is also nearly certain to play fundamental roles in cardiac fibroblasts that deposit extracellular matrix, immune cells (e.g. macrophages) that mediate inflammation in the injured heart, and other cardiac cell types, although this review will focus on established functions of palmitoylation in cardiomyocytes and future areas of investigation.

Palmitoylation is catalyzed by a family of 23 zinc finger and Asp-His-His-Cys domain-containing S-acyltransferases or zDHHC enzymes and reversed by acyl protein thioesterases of the lysophospholipase (*LYPLA1*, *LYPLA2*, and *LYPLAL1* encoding Acyl Protein Thioesterase 1, 2, and -like-1, respectively) and α/β-hydroxylase domain-containing families (ABHD17A/B/C and ABHD10) [2,3,10–13]. A majority of zDHHCs localize to intracellular membranes, predominantly the Golgi apparatus but also endoplasmic reticulum (ER) and intracellular vesicles, with a couple also found at the sarcolemma [1–3,14]. zDHHCs are 4- or 6-pass transmembrane proteins with divergent N- and C-termini and their highly conserved enzymatic DHHC domains in the cytosolic loop [14–17]. As such, substrates are modified by zDHHCs on cytosolic or juxtamembrane cysteines to facilitate membrane association that alters trafficking, localization, structure, and/or function [1,17]. In contrast, the depalmitoylases, with the exception of ABHD10 that is found in mitochondria [18], are soluble cytoplasmic proteins [3,13], although APT1 and APT2 can themselves be targeted to cellular membranes by cysteine palmitoylation [19–21].

## Palmitoylating and depalmitoylating enzymes in the heart

Although a majority of studies investigating zDHHC enzymes have focused on their functions in the context of neurology and several zDHHCs do indeed have central nervous system (CNS)-enriched expression, many zDHHCs are expressed and even enriched in cardiomyocytes [22–25]. In contrast, depalmitoylating thioesterases are rather ubiquitously expressed, including in the heart [23,26]. Transcript levels of genes encoding nodal Golgi enzymes (e.g. zDHHC3 and zDHHC7) are reported to be particularly abundant in the postnatal human and rodent heart [22,27]. However, difficulties in extraction and antibody-based detection of native zDHHC proteins have made reliable determination of zDHHC protein expression levels in the healthy and diseased heart challenging [25]. Notably, transcript levels of many palmitoylating and depalmitoylating enzymes were found to be dysregulated (mostly down-regulated) in the myocardium of human heart failure patients [23]. Cardiac protein levels of the sarcolemmal S-acyltransferase, zDHHC5, and the Golgi enzymes zDHHC3 and zDHHC7 are significantly increased in the murine heart after 8 weeks of pressure overload-induced hypertrophy [23,24], suggesting a role for these enzymes and palmitoylation of their substrates in adaptation to cardiac stress. However, zDHHC5 levels are reduced in a porcine model of ischemia-reperfusion injury and in human ischemic heart failure [23], highlighting the potential for distinct and dynamic regulation of the expression of palmitoylation machinery in response to different pathological stimuli. Moreover, the potential for stimulus-dependent modulation of zDHHC enzyme activity, such as through post-translational modifications, and the lack of methodologies to quantify the activity of specific S-acyltransferases *in vivo*, makes it challenging to ascertain enzyme-substrate regulation in the context of cardiac pathophysiology. Additional gain- and loss-of-function animal models are needed to fully elucidate the functions of palmitoylation, zDHHCs, and depalmitoylating enzymes in cardiac physiology and stress adaptation.

Despite the limited number of studies investigating palmitoylation in cardiomyocytes *in vivo*, gene-targeted and transgenic mouse models have already uncovered indispensable functions of zDHHC enzymes in the heart. Genetic ablation of the ER-localized enzyme zDHHC16 results in perinatal lethality caused by cardiac developmental defects, including severe bradycardia and cardiomyocyte nuclear dysmorphology, in addition to abnormal eye development [28]. In contrast, myocardium from *Zdhhc5* gene-deleted mice exhibits more effective recovery of contractile force following anoxia [29]. An *in vivo* gain-of-function screen with adeno-associated virus 9 (AAV9)-mediated overexpression of select zDHHC enzymes in the heart found no overt cardiac phenotype from overexpression of the sarcolemmal enzyme zDHHC5, Golgi-localized zDHHC13, or the ER enzyme zDHHC6, whereas overexpression of the closely related Golgi enzymes zDHHC3 or zDHHC7 caused severe lethal dilated cardiomyopathy [24]. Cardiomyocyte-specific transgenic mice overexpressing zDHHC3 recapitulate this phenotype, exhibiting severe dilated cardiomyopathy and bradycardia followed by lethality around 6 weeks of age when zDHHC3 is overexpressed from around birth in ventricular cardiomyocytes (α-myosin heavy chain (MHC) promoter-driven expression) [24]. However, when initiation of zDHHC3 transgene expression is delayed until young adulthood, it does not impact heart rate but results in congestive heart failure including peripheral edema, dyspnea, and cardiac hypertrophy [24]. Notably, transgenic mice with cardiomyocyte-specific overexpression of an enzymatically dead mutant of zDHHC3 do not exhibit any discernable cardiac phenotype [24], indicating that cardiac dysfunction and maladaptive remodeling evoked by zDHHC3 overexpression is dependent on its S-acyltransferase activity. Transgenic mice with cardiomyocyte-specific overexpression of the Golgi enzyme, zDHHC9, exhibit normal cardiac function in young adulthood but develop dilated cardiomyopathy with advanced age [5], albeit relatively mild compared with overexpression of zDHHC3.

The relatively few studies interrogating zDHHC enzymes in cardiac myocytes in animal models have revealed essential functions in cardiac development, homeostasis, and disease pathogenesis. Future studies in mouse models with conditional deletion of zDHHC enzymes and depalmitoylating thioesterases in cardiomyocytes are needed to elucidate palmitoylation-regulated signaling and intracellular trafficking mechanisms that participate in cardiac disease pathogenesis. Palmitoylation has well-established roles in regulating cardiac electrophysiology through modulation of ion channel trafficking and activity (Table 1), which has been extensively reviewed elsewhere [1,4,14]. Here, we review what is currently known about the roles of protein palmitoylation in cardiomyocytes, focusing on its roles in the regulation of intracellular signaling, protein trafficking, and regulation of exocytosis and cardiac endocrinology (Table 1).

**Table 1.** Protein substrates for which critical roles of cysteine palmitoylation have been established in cardiomyocytes

Target	zDHHC(s)	Site(s)	Effect(s)	Refs.
*Electrophysiology and calcium cycling*				
PLM	zDHHC5	Cys-40*	Inhibits Na^+^/K^+^-ATPase sodium pump activity	[30]
NCX1	zDHHC5	Cys-739	Promotes XIP-induced inactivation of NCX1	[31,32]
Jph2	–	Cys-15, 29, 328, 678	Stabilizes endoplasmic/sarcoplasmic reticulum–plasma membrane junctions	[33]
PLN	zDHHC16	Cys-36*	Inactivation of PLN and augmented SERCA2a pump activity	[28]
Ca_v_1.2	–	Cys-136, 519, 543*	Regulates voltage sensitivity of channel	[34]
Na_v_1.5	–	Cys-981*	Regulates channel gating	[35]
KChIP2	–	Cys-45, 46*	Plasma membrane/sarcolemmal localization	[36]
*Cardiomyocyte signal transduction*				
Gα_s_, Gα_i_	zDHHC5	–	Regulates cAMP levels	[52]
β2-adrenergic receptor	–	Cys-341*	Regulates receptor internalization, cAMP-PKA signaling	[37,51]
sAC	–	Cys-342*	Increased cAMP production and Rap1 activation	[53]
Rac1	zDHHC3	Cys-178	Plasma membrane localization and GTP loading	[24,49]
STING	–	Cys-91	Activates downstream IRF3 signaling	[74]
*Cardiomyocyte exocytosis and endocytosis*				
Rab3gap1	zDHHC9	–	Impaired Rab3 GTPase cycling and ANP release	[5]
Membrane proteins				
(PLM, flotillin-2)	zDHHC5	–	Massive endocytosis (MEND)	[29]

Palmitoylated proteins in cardiomyocytes as well the zDHHC S-acyltransferases modifying them, modified cysteine(s), and cellular effect are indicated.

zDHHC, zinc finger and Asp-His-His-Cys domain containing; PLM, phospholemman, NCX1, sodium–calcium exchanger 1; XIP, exchange inhibitory peptide; Jph2, junctophilin-2; PLN, phospholamban; SERCA2a, sarcoendoplasmic reticulum ATPase 2a; KChIP2, potassium voltage-gated channel interacting protein 2; PKA, protein kinase A; sAC, soluble adenylyl cyclase; Rap1, Ras-related protein 1; STING, stimulator of interferon genes; IRF3, interferon regulatory factor 3; Rac1, Ras-related C3 botulinum toxin substrate 1; Rab3gap1, Rab3 GTPase activating protein 1; ANP, atrial natriuretic peptide; MEND: massive endocytosis. *Asterisks indicate that site-directed mutagenesis or functional effects of mutants of the listed cysteine residues have been assessed in either primary or iPSC-derived cardiomyocytes.

## Electrophysiology and calcium cycling

Palmitoylation has been most extensively studied in cardiomyocytes in the context of regulation of ion channels, transporters, and exchangers that modulate the cardiomyocyte action potential and calcium handling [1,4,36]. Indeed, palmitoylation of the pore-forming subunits of cardiac voltage-gated sodium (Na_v_1.5) and calcium (Ca_v_1.2) channels modulates channel current and voltage sensitivity, respectively [34,35]. Notably, the sarcolemmal S-acyltransferase, zDHHC5, is particularly instrumental in modulating cardiomyocyte membrane potential and ion transport across the plasma membrane. The cardiac sodium pump (Na^+^/K^+^-ATPase) is palmitoylated on its enzymatic α and β subunits but, more significantly, undergoes dynamic regulation of its activity by zDHHC5-catalyzed palmitoylation of its accessory regulatory subunit, phospholemman (PLM) [22,30]. The activity of the sodium–calcium exchanger 1 (NCX1) in cardiomyocytes is also intimately regulated by zDHHC5-mediated palmitoylation [31,32,43] and has been extensively reviewed elsewhere [1,14], highlighting the complex and diverse mechanisms by which palmitoylation can alter cardiomyocyte excitability.

Excitation–contraction coupling and sarcoplasmic reticulum (SR) calcium cycling, in addition to being impacted by palmitoylation-dependent modulation of the cardiomyocyte action potential, are also directly regulated by palmitoylation. Trafficking and membrane association of junctophilin-2 (Jph2), which is critical for the maintenance of the structure of the dyad and effective excitation–contraction coupling [44–47], are regulated by palmitoylation [33], which could indirectly impact intracellular calcium cycling in cardiomyocytes. In addition, SR calcium cycling can be more directly modulated by post-translational cysteine palmitoylation. Phospholamban (PLN), which inhibits the activity of sarcoendoplasmic reticulum ATPase 2a (SERCA2a) to modulate SR calcium reuptake, is palmitoylated by zDHHC16 at cysteine-36 [28]. Hearts lacking zDHHC16 exhibited reduced palmitoylation of PLN that was associated with increased interaction of PLN with protein phosphatase 1α (PP1α) and reduced inhibitory phosphorylation of PLN at serine-16 [28]. This suggests that palmitoylation of PLN promotes its dephosphorylation/inactivation and may serve to enhance SERCA2a pump activity, although SR calcium cycling was not directly examined in *Zdhhc16*-deleted cardiomyocytes.

It was recently discovered that the pore-forming α1C subunit of the L-type calcium channel (Ca_v_1.2) is palmitoylated in ventricular cardiomyocytes [34]. Comprehensive biochemical and biophysical characterization in HEK cells identified cysteine-136 within the N-terminus and cysteines-519 and -543 in the domain I–II linker region of the α1C subunit as the functionally modified residues impacting the voltage dependence of Ca_v_1.2 current [34]. Calcium transient amplitudes were reduced in human induced pluripotent stem cell-derived cardiomyocytes expressing the palmitoylation-deficient α1C^C136/519/543A^ mutant [34], supporting a role for α1C palmitoylation in regulating Ca_v_1.2 activity in cardiomyocytes. Thus, the duration and magnitude of elevation of cytosolic calcium levels during systole are controlled by palmitoylation of multiple calcium handling and regulatory proteins, providing multiple mechanisms by which palmitoylation can impact myofilament contraction downstream of alterations of cardiomyocyte electrophysiology.

## Cardiomyocyte signal transduction

Intracellular signal transduction occurs predominantly through a series of transient protein–protein interactions and enzymatic activities (e.g. catalysis of post-translational modifications) at cellular membranes that initiate changes in protein structure and function and a cascade of activation of downstream effectors that ultimately elicits a cellular physiologic response. Consequently, the membrane microdomain localization, topography, and/or activity of a myriad of signaling molecules, including membrane-localized receptors and soluble cytosolic signal transducing proteins that transiently associate with cellular membranes, are controlled by dynamic cysteine palmitoylation. For instance, many G protein-coupled receptors (GPCRs) are palmitoylated on their C-terminal cytosolic tail, a majority of heterotrimeric Gα subunits are palmitoylated on their N-terminus, and some small GTPases have cysteines adjacent to the hypervariable region that undergo palmitoylation cycling (e.g. H-Ras, N-Ras) to dynamically control signaling output [1,41,48–50]. Palmitoylation is an optimal mechanism to rapidly activate or impart spatiotemporal control to intracellular signal transduction in cardiomyocytes and profoundly influence cardiac function and pathophysiology. Nonetheless, there has been limited exploration of palmitoylation-dependent regulation of intracellular signaling in cardiac myocytes *in vivo* or in a pathophysiologic context.

### G protein signaling

**Figure 1.**
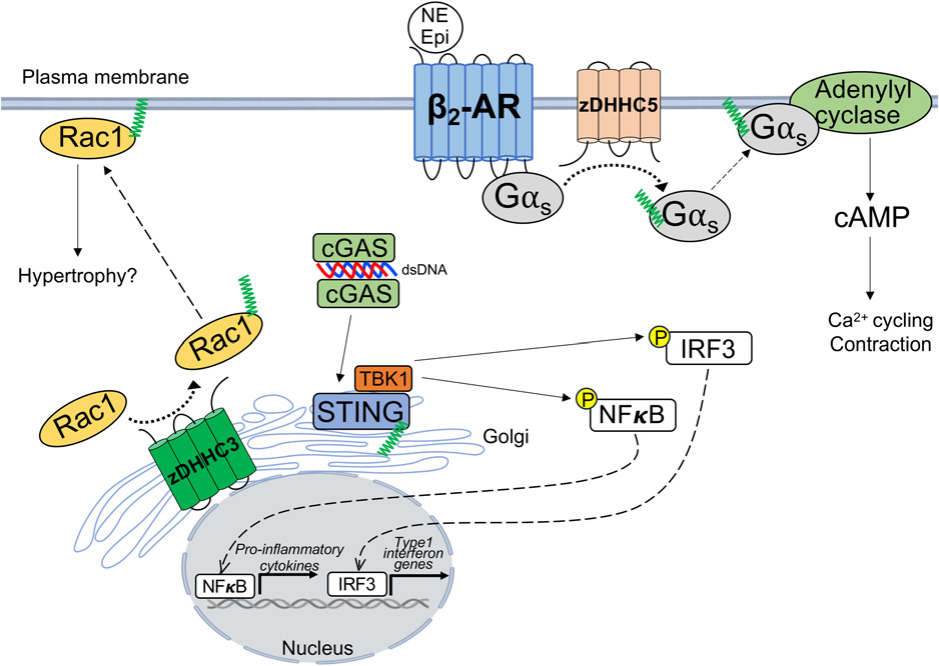
Regulation of cardiomyocyte signal transduction by protein palmitoylation. zDHHC3-mediated palmitoylation of Rac1 promotes sarcolemmal targeting and activation of Rac1. STING palmitoylation at the Golgi is triggered by binding of cGAS to cytosolic DNA and necessary for downstream nuclear translocation and transcriptional activities of IRF3 and NFκB. zDHHC5 promotes palmitoylation of Gαs and downstream induction of cAMP and cytosolic calcium in response to stimulation of the β_2_-adrenergic receptor. Rac1, Ras-related C3 botulinum toxin substrate 1; cGAS, cyclic GMP–AMP synthase; STING, stimulator of interferon genes; TBK1, TANK-binding kinase 1; NFκB, nuclear factor kappa B; IRF3, Interferon regulatory factor 3; β_2_-AR, β_2_-adrenergic receptor; NE, norepinephrine; Epi, epinephrine; dsDNA, double-stranded deoxyribonucleic acid; Gαs, G_s_ alpha subunit; cAMP, cyclic adenosine monophosphate.

**Figure 2.**
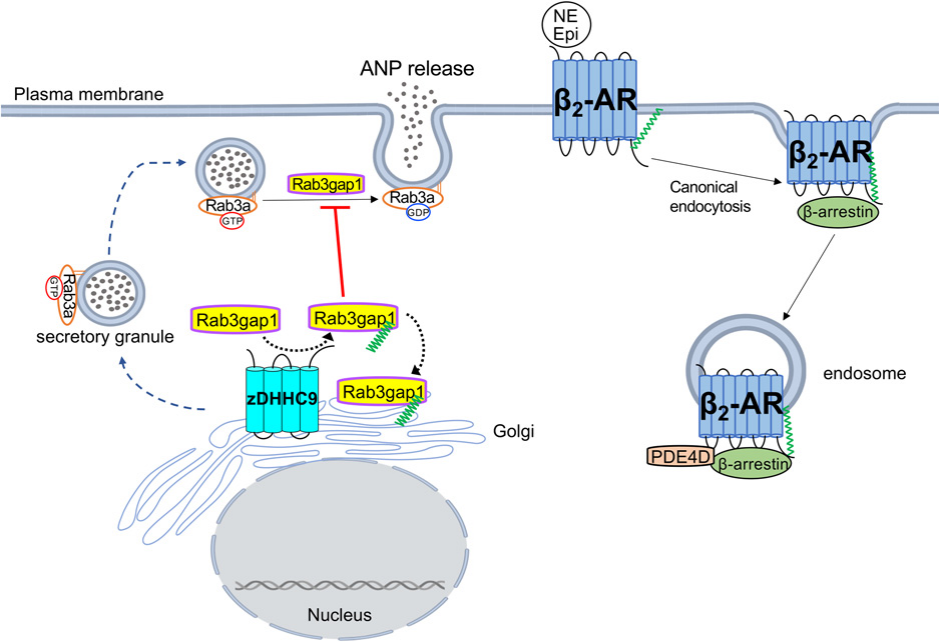
Regulation of cardiomyocyte exocytosis and endocytosis by protein palmitoylation. zDHHC9 promotes palmitoylation and increased Golgi retention of Rab3gap1 in cardiomyocytes resulting in impaired Rab3a nucleotide cycling and a deficit in ANP secretion. Palmitoylation of the C-terminal tail of the β_2_-AR is required for internalization via canonical β-arrestin-mediated endocytosis and for its association with PDE4D to terminate compartmentalized endosomal cAMP signaling by internalized β_2_-ARs following agonist stimulation. Rab3gap1, Rab3 GTPase activating protein 1; ANP, atrial natriuretic peptide; Rab3, Ras-related protein 3; β_2_-AR, β_2_-adrenergic receptor; PDE4D, phosphodiesterase 4D.

Palmitoylation is an important determinant of β-adrenergic signaling in cardiomyocytes. Many GPCRs including β-adrenergic receptors are themselves regulated by palmitoylation [38,51] but notably, it has been demonstrated that Gαs and Gαi are palmitoylated in cardiomyocytes in response to isoproterenol (Figure 1). Knockdown of zDHHC5 mitigated induction of Gαs and Gαi palmitoylation, cAMP levels, and calcium transient frequency in neonatal cardiac myocytes in response to β-adrenergic stimulation [52]. The β_2_-adrenergic receptor (β_2_-AR) is palmitoylated in cardiomyocytes on cysteine-341 in its C-terminal cytosolic tail, where many GPCRs are canonically regulated by palmitoylation [1,37–39,51]. Palmitoylation of the β_2_-AR in cardiomyocytes appears to be essential for the termination of cAMP signaling by internalized β_2_-ARs [51]. Expression of a palmitoylation-deficient β_2_-AR^C341A^ mutant in neonatal mouse cardiomyocytes lacking both β_1_-AR and β_2_-AR resulted in normal expression at the sarcolemma but defective interaction with β-arrestin 2 and phosphodiesterase 4D (PDE4D) and elevated cytosolic cAMP levels and PKA activity in response to isoproterenol [51], suggesting that palmitoylation is required for recruitment of PDE4D to β_2_-AR at endosomes following β-arrestin-dependent internalization (Figure 2). The β_2_-AR has also been shown to be palmitoylated at cysteine-265 within its third intracellular loop by the Golgi enzymes zDHHC9/14/18 in response to agonist stimulation that impacts its intracellular trafficking and cell surface expression [38], but whether this regulatory mechanism occurs in cardiomyocytes has not been tested. Moreover, soluble adenylyl cyclase (sAC) is palmitoylated in cardiomyocytes at cysteine-342 in response to high palmitate concentrations to evoke cAMP production and activation of the small GTPase Rap1 [53], highlighting palmitoylation-dependent control of not just GPCRs and Gα subunits, but also cross-talk and compartmentalization of second messenger signaling. Further research is needed to understand the complex regulation of adrenergic receptor signaling by palmitoylation, including the distinct mechanisms by which palmitoylation modulates β_1_-AR and β_2_-AR signaling from the sarcolemma and intracellular compartments, impacts on compartmentalized cAMP production and effector signaling, and ultimately how this modulates cardiomyocyte calcium handling and contractility.

Perhaps the best-characterized example of regulation of intracellular signal transduction by protein palmitoylation involves palmitoylation cycling on H-Ras and N-Ras that are necessary for membrane translocation and sustained signaling outputs [10,50]. Indeed, genetic or pharmacological inhibition of H/N-Ras palmitoylation or depalmitoylation impairs palmitoylation cycling and disrupts cancer cell growth and proliferation [10,40,50]. In addition to H-Ras and N-Ras, other small GTPases of the Ras superfamily undergo palmitoylation on their C-terminus, along with the canonical irreversible prenylation on the cysteine on the CAAX motif of the ultimate C-terminus [10,41,50]. Ras-related C3 botulinum toxin substrate 1 (Rac1) is a small GTPase within the Rho family with instrumental roles in regulating the actin cytoskeleton as well as inducing the catalytic activity of NADPH oxidase-2 (Nox2) to evoke superoxide production and oxidative stress [54–57]. Rac1 is palmitoylated at cysteine-178 on its C-terminus to promote its activation, localization to lipid rafts, and cell migration in immortalized cell lines [24,49]. Proteomic studies identified Rac1 as a novel substrate of the Golgi-localized enzyme, zDHHC3, and subsequent studies found that cardiomyocyte-specific overexpression of zDHHC3 increased Rac1 palmitoylation in the heart [24]. zDHHC3 overexpression also elicited robust enhancement of Rac1 activity and translocation to the sarcolemma (Figure 1), along with induction of the protein levels of all Rho family small GTPases, all of which preceded heart failure in zDHHC3 transgenic mice [24]. These studies demonstrate a critical role for palmitoylation of soluble signaling proteins at the surface of the cardiomyocyte Golgi in the regulation of intracellular signaling, including the promotion of pathogenic signaling by small GTPases associated with cardiac maladaptation and heart failure [24,58,59]. Further investigation is needed to determine the role of palmitoylated Rac1 in cardiomyocytes, including if there are differential effectors and functions of palmitoylated Rac1 versus depalmitoylated Rac1 (which can still associate with membranes via its C-terminal polybasic domain and geranylgeranyl lipidation) and if Rac1-dependent Nox2 activity, cardiac hypertrophy and oxidative stress [60] require its palmitoylation.

### cGAS-STING signaling

Owed to the current lack of S-acyltransferase inhibitors with specificity for particular zDHHC enzymes, perhaps the most tractable pharmacological strategy to target protein palmitoylation to date has been the development of small molecules that alkylate substrate cysteine residues to prevent their modification by palmitoylation [1,61–63]. Indeed, modulation of the innate immune response and type I interferon signaling by palmitoylation of stimulator of interferon genes (STING) has been harnessed clinically through the development of small molecules that covalently bind to cysteine residues on STING that need to be palmitoylated for its proper activation. STING palmitoylation at the Golgi is required for its oligomerization, formation of protein signaling complexes, and activation of downstream effectors to ultimately induce a host defense gene program through the transcriptional activities of interferon regulatory factor 3 (IRF3) and nuclear factor kappa B (NFκB) [61–68] (Figure 1). Cyclic GMP–AMP synthase (cGAS) senses cytosolic DNA that originates from bacterial or viral infection or self-DNA released from mitochondria or dying cells and in turn generates 2′,3′-cyclic GMP–AMP (cGAMP) that binds to STING and evokes its palmitoylation, activation, and downstream production of type I interferons as an integral cellular mechanism driving the innate immune response [64,65,69]. High-throughput screening of inhibitors of interferon signaling identified multiple nitrofuran derivatives that alkylate the essential palmitoylated cysteine-91 residue of STING [61]. Notably, nitro fatty acids, which are protective in cardiovascular disease [70,71], also alkylate STING to prevent its activation [64]. Small molecule inhibitors of STING palmitoylation are being pursued for the treatment of autoinflammatory diseases including lupus erythematosus and psoriasis [62,63,68] and phase II clinical trials have been conducted for the treatment of focal segmental glomerulosclerosis with nitro fatty acids [62].

With regards to cardiac pathophysiology, the cGAS-STING pathway plays an essential role in cardiac inflammation after myocardial infarction by activating an IRF3-dependent interferon-stimulated gene program in response to sensing of DNA from dying or damaged cardiomyocytes/mitochondria [72,73]. This promotes monocyte recruitment and macrophage activation in the heart, inflammatory cytokine expression, deterioration of cardiac function, and mortality, all of which can be ameliorated by genetic ablation of cGAS or IRF3 [72,73]. Notably, pharmacological inhibition of STING activation with a small molecule inhibitor of STING palmitoylation reduces adverse cardiac remodeling and dysfunction in a mouse model of chronic kidney disease [74] and nitro fatty acid treatment improves cardiac function and reduces myocardial fibrosis in a genetic mouse model of dilated cardiomyopathy [42]. In addition to impairing cGAS-STING signaling and interferon production in macrophages to dampen maladaptive myocardial inflammation [72], the protective effects of inhibiting STING palmitoylation and activation in the heart also arise at least in part from its effects in cardiomyocytes and repressing STING-dependent cardiomyocyte pyroptosis [75,76] as cardiomyocyte-specific deletion of STING ameliorates cardiac hypertrophy and systolic dysfunction in response to chronic kidney injury [74] and knockdown of STING expression in cardiomyocytes restores cardiac function and dampens myocardial inflammation in diabetic cardiomyopathy [75]. Thus, targeting palmitoylation-dependent activation of the cGAS-STING signaling pathway in cardiomyocytes has the potential to mitigate cardiac remodeling in ischemic and non-ischemic heart diseases associated with adverse myocardial inflammation.

## Cardiomyocyte exocytosis and endocytosis

The abundance of zDHHC enzymes localized at the ER, Golgi, and endomembranes and the inherent lipophilicity imparted by conjugation of a fatty acid onto a protein makes palmitoylation ideally suited to regulate the flux and anterograde trafficking of peripheral membrane proteins through the secretory pathway to their ultimate plasmalemmal or organellar cellular membrane destination [77–79]. Indeed, regulated palmitoylation is an essential mechanism that can modulate intracellular protein trafficking through multiple mechanisms and consequently has profound impacts on cardiomyocyte signal transduction and electrophysiology (see above). Palmitoylation can regulate the trafficking and recycling of not just membrane proteins themselves, but also the molecular machinery and regulatory proteins controlling vectorial transport through the endomembrane system.

Palmitoylation has essential functions in the regulation of exocytic release of hormones, peptides, and neurotransmitters from secretory vesicles in specialized cell types [80–85]. A palmitoylation-dependent pathway governing sarcolemmal delivery of secretory vesicles and natriuretic peptide secretion by cardiomyocytes was recently uncovered [5]. Proteomic studies identified Rab3 GTPase activating protein 1 (Rab3gap1) as a substrate of zDHHC9-mediated palmitoylation in cardiomyocytes. Rab3gap1 is the dedicated GTPase activating protein (GAP) that inactivates Rab3, a small GTPase primarily involved in exocytosis, to its GDP-bound state necessary for its dissociation from secretory vesicles to reinitiate the GTPase cycle (Figure 2) [86–88]. Palmitoylation of Rab3gap1 by zDHHC9 at the cardiomyocyte Golgi membrane resulted in Golgi retention of Rab3gap1 and repression of cellular GAP activity on Rab3 and consequently higher levels of active Rab3-GTP [5]. zDHHC9-mediated palmitoylation promoted the movement of Rab3 from the Golgi to peripheral secretory vesicles in cardiomyocytes, but this was accompanied by a deficit in the release of atrial natriuretic peptide (ANP) due to impairment of the Rab3 GTPase cycle [5]. ANP secretion is induced in cardiomyocytes in response to cardiac stress such as neurohumoral stimulation (increased circulating angiotensin-II, catecholamines) and increased blood volume (atrial distension) as an endocrine mechanism of cardiovascular protection that increases vasodilation, natriuresis, and diuresis to reduce blood pressure and cardiac workload [89–92]. Pathophysiologic stimulation of ANP secretion in cardiomyocytes with phenylephrine elicited robust release of ANP as expected, which was associated with enhanced Rab3 activity and localization of ANP to Rab3-positive peripheral secretory vesicles [5]. Importantly, phenylephrine treatment enhanced Rab3gap1 palmitoylation in cardiomyocytes and knockdown of zDHHC9 expression prevented Rab3gap1 palmitoylation and Rab3-GTP loading in response to phenylephrine but promoted even greater release of ANP [5]. A majority of ANP secreted by the diseased heart originates from atrial myocytes that contain specialized atrial granules loaded with processed ANP poised for exocytic release through a regulated secretory pathway [92–99] compared with the constitutive secretory pathway in neonatal ventricular cardiomyocytes in which a majority of the mechanistic studies of zDHHC9 and Rab3gap1 palmitoylation on ANP release were performed. Thus, this palmitoylation-dependent mechanism regulating cardiomyocyte exocytosis warrants further investigation in atrial cardiomyocytes that are by far the predominant source of the vast majority of natriuretic peptides released into the circulation. It is however noteworthy that pharmacological elevation of circulating natriuretic peptides by neprilysin inhibitor treatment, in combination with an angiotensin receptor antagonist, is efficacious and becoming widely used for the treatment of heart failure with reduced ejection fraction (HFrEF) [100,101]. Thus, inhibition of zDHHC9 activity or palmitoylation of Rab3gap1 could potentially stimulate greater release and consequently greater circulating levels of natriuretic peptides in response to increased neurohumoral stimulation that occurs in cardiovascular disease, which may have therapeutic implications for the treatment of heart failure and/or hypertension.

Palmitoylation also plays an important role in controlling endocytosis to facilitate the internalization of receptors, turnover of plasma membrane domains, and/or phagocytosis of extracellular contents [102–104]. Cardiomyocytes, like other cell types, possess heterogenous endocytic pathways to enable internalization and vesicular delivery of proteins, plasma membranes, and/or extracellular components to distinct intracellular compartments. As described above, palmitoylation of the β_2_-AR in cardiomyocytes is essential for the cessation of compartmentalized cAMP production evoked by internalized receptors presumably at endosomes (Figure 2). Mutation of the β_2_-AR palmitoylation site causes aberrant internalization through a caveolin-dependent pathway rather than canonical β-arrestin-dependent endocytosis and elevation of cytosolic cAMP levels in response to isoproterenol [51], although the dynamics of β_2_-AR palmitoylation/depalmitoylation in its endocytic recycling and compartmentalized signaling are not fully understood. In cardiomyocytes exposed to anoxia and reoxygenation, mitochondrial damage and mitochondrial permeability transition pore opening result in massive endocytosis (MEND) in which a large portion of the plasma membrane is internalized in a process that requires the S-acyltransferase activity of the sarcolemmal enzyme zDHHC5 [29]. Genetic ablation of zDHHC5 blunts the anoxia-reoxygenation MEND response in cardiomyocytes and improves contractility of reoxygenated myocardium [29], suggesting zDHHC5-regulated MEND and sarcolemmal turnover play fundamental roles in cardiac homeostasis and stress adaptation. Palmitoylation undoubtedly has additional functions in controlling endocytic pathways in cardiac myocytes, but more studies are needed to uncover mechanisms, targets, and physiologic consequences.

## Future directions

Many of the mechanistic and biochemical studies assessing functions of palmitoylation and enzymatic regulation by zDHHC enzymes have been performed in immortalized cell lines (e.g. HEK cells) and not in primary cardiomyocytes or *in vivo*. Although many of these findings do recapitulate the *in vivo* enzyme regulation and impacts on substrate functions, there are many examples of cell type-specific regulation of substrate palmitoylation, particularly in highly specialized cells with unique membrane domains (e.g. cardiomyocytes, neurons) [33,80,105–107]. Moreover, many proteins regulated by palmitoylation are uniquely expressed or have distinct functions in cardiac myocytes that necessitate cell type-specific regulatory mechanisms to direct dynamic association with intracellular membrane microdomains. Furthermore, the morphogenetically distinct cytoarchitecture of cardiomyocytes and even the heterogeneity in subcellular organelle morphology and distribution of membrane domains amongst atrial versus ventricular cardiomyocytes present a distinct intracellular membrane environment and likely unique zDHHC-dependent mechanisms of regulation of protein substrate trafficking, membrane targeting, and function. The sarcolemmal enzyme, zDHHC5, has received a lot of attention with regards to enzymatic regulation of palmitoylation in cardiomyocytes, but other zDHHCs including Golgi-localized enzymes play fundamental roles in coordinating intracellular trafficking and secretory pathway activity as well as dynamic targeting and modulation of compartmentalized signaling in cardiomyocytes. Indeed, the palmitoylation machinery (zDHHCs and depalmitoylases) is optimally positioned to fine-tune protein trafficking in the context of the distinct intracellular membrane topography of cardiac myocytes, including targeting proteins to specific sarcolemmal microdomains such as the T-tubule, intercalated disc, and costamere. However, to date, there have been limited investigations of zDHHC enzymes and the functions of palmitoylation in the context of cardiac physiology and disease. Future *in vivo* studies with cardiomyocyte-specific genetic manipulation of palmitoylating and depalmitoylating enzymes and substrate palmitoylation sites will facilitate translating biochemical and molecular discoveries of protein palmitoylation in cardiomyocyte biology to the physiology and pathophysiology of cardiac homeostasis and disease.

## Perspectives

- Palmitoylation has essential functions in cardiac physiology through the regulation of intracellular trafficking, secretory pathway function, and signal transduction in cardiomyocytes.
- The unique plasma membrane and organellar membrane landscape of cardiac myocytes make dynamic protein palmitoylation an ideal post-translational mechanism to impart regulatory control over protein trafficking and compartmentalization of intracellular signal transduction in this highly specialized cell type.
- Future studies in animal models and primary cardiomyocytes with genetic and pharmacologic manipulation of zDHHC enzymes, depalmitoylases, and substrate palmitoylation sites will help uncover mechanisms by which palmitoylation controls cardiomyocyte signaling, protein trafficking, and electrophysiology in the context of cardiac homeostasis and in cardiac pathologies such as myocardial ischemia, cardiac hypertrophy, and heart failure.

## Open Access

Open access for this article was enabled by the participation of the University of Michigan in an all-inclusive *Read & Publish* agreement with Portland Press and the Biochemical Society under a transformative agreement with UM.

## Abbreviations

β2-AR: beta-2 adrenergic receptor
ABHD: α/β-hydroxylase domain containing
ANP: atrial natriuretic peptide
cGAMP: 2′,3′-cyclic GMP–AMP
cGAS: cyclic GMP–AMP synthase
GAP: GTPase activating protein
GPCR: G protein coupled receptor
HFrEF: heart failure with reduced ejection fraction
IRF3: interferon regulatory factor 3
Jph2: junctophilin-2
KChIP2: potassium channel interacting protein 2
LYPLA: lysophospholipase
MEND: massive endocytosis
NCX1: sodium–calcium exchanger 1
NFκB: nuclear factor kappa B
Nox2: NADPH oxidase-2
PDE4D: phosphodiesterase 4D
PLM: phospholemman
PLN: phospholamban
Rab3gap1: Rab3 GTPase activating protein 1
Rac1: Ras-related C3 botulinum toxin substrate 1
sAC: soluble adenylyl cyclase
SERCA2a: sarcoendoplasmic reticulum ATPase 2a
SR: sarcoplasmic reticulum
STING: stimulator of interferon genes
zDHHC: zinc finger and Asp-His-His-Cys domain containing
